# MHC Haplotype Matching for Unrelated Hematopoietic Cell Transplantation

**DOI:** 10.1371/journal.pmed.0040008

**Published:** 2007-01-30

**Authors:** Effie W Petersdorf, Mari Malkki, Ted A Gooley, Paul J Martin, Zhen Guo

**Affiliations:** 1 Division of Clinical Research, Fred Hutchinson Cancer Research Center, Seattle, Washington, United States of America; 2 The University of Washington School of Medicine, Seattle, Washington, United States of America; 3 The Department of Biostatistics, University of Washington, Seattle, Washington, United States of America; University of Cambridge, United Kingdom

## Abstract

**Background:**

Current criteria for the selection of unrelated donors for hematopoietic cell transplantation (HCT) include matching for the alleles of each human leukocyte antigen (HLA) locus within the major histocompatibility complex (MHC). Graft-versus-host disease (GVHD), however, remains a significant and potentially life-threatening complication even after HLA-identical unrelated HCT. The MHC harbors more than 400 genes, but the total number of transplantation antigens is unknown. Genes that influence transplantation outcome could be identified by using linkage disequilibrium (LD)-mapping approaches, if the extended MHC haplotypes of the unrelated donor and recipient could be defined.

**Methods and Findings:**

We isolated DNA strands extending across 2 million base pairs of the MHC to determine the physical linkage of *HLA-A, -B,* and *-DRB1* alleles in 246 HCT recipients and their *HLA-A, -B, -C, -DRB1, -DQB1* allele-matched unrelated donors. MHC haplotype mismatching was associated with a statistically significantly increased risk of severe acute GVHD (odds ratio 4.51; 95% confidence interval [CI], 2.34–8.70, *p* < 0.0001) and with lower risk of disease recurrence (hazard ratio 0.45; 95% CI, 0.22–0.92, *p* = 0.03).

**Conclusions:**

The MHC harbors genes that encode unidentified transplantation antigens. The three-locus *HLA-A, -B, -DRB1* haplotype serves as a proxy for GVHD risk among HLA-identical transplant recipients. The phasing method provides an approach for mapping novel MHC-linked transplantation determinants and a means to decrease GVHD-related morbidity after HCT from unrelated donors.

## Abstract 2

A novel method of MHC haplotype matching provides a means to decrease graft-versus-host disease-related morbidity after transplantation from unrelated donors.

## Editors' Summary

**Background.:**

Graft rejection and graft-versus-host disease (GVHD) are feared complications of hematopoietic cell transplantation (HCT). GVHD can affect all parts of the body, and, if severe (grade III to IV out of a scale of IV), can lead to the death of the transplant recipient. GVHD or rejection of the graft occurs when there are differences in specific proteins involved in the immune response (known as HLA antigens) between donor and recipient that stimulate the immune reaction. GVHD and graft rejection occur most often in people who receive transplants from unrelated donors because, although when donors are matched to recipients matching is done for the most important HLA antigens known to be involved, it has not technically been possible to match for all possible antigens. However, the human genome is organized into segments or blocks of closely linked genetic variants that are inherited as “haplotypes” on the same DNA strand of a chromosome. Most of the genes that code for HLA antigens are physically located together in one part of the human genome, known as the MHC region. Currently three HLA markers from this region (HLA-A, -B, -DRB1) are matched when matching donors and recipients. If it were possible to better map the structure of this region, it would be possible to better match recipients and donors (especially unrelated donors) for the unidentified transplantation antigens and reduce the chance of recipients getting GVHD or rejecting their grafts.

**Why Was This Study Done?:**

Current strategies to define MHC haplotype blocks look at, on average, a length of only 18,000 base pairs and hence cannot define extended MHC haplotypes. Previously, this group of researchers developed a method of defining the *HLA-A, B, DR* haplotypes in recipients and their HLA-matched unrelated transplant donors using high-quality DNA containing 2 million base pairs across the MHC region. They wanted see if using this technique might provide a way to better assess the risk recipients have of developing GVHD or of having recurrent disease.

**What Did the Researchers Do and Find?:**

They studied 246 HCT recipients and their donors who had been matched for HLA-A, -B, -C, -DRB1, -DQB1 by current techniques. The recipients were having HCT for a variety of hematological cancers: acute lymphoid leukemia, acute myeloid leukemia, chronic myeloid leukemia, or myelodysplastic syndrome. They found that, using the new technique, 22% of the donor–recipient pairs were haplotype-mismatched. Taking various other factors into account, including age, and patient and donor gender, MHC haplotype mismatching was associated with an approximately four times greater risk of severe acute GVHD but with a lower risk of disease recurrence. The lower risk of recurrence is believed to be because transplanted cells do not only replace abnormal cancerous cells but also react against them and therefore decrease the chance of the cancer recurring; mismatched cells are known to be more stimulated to react against the cancerous cells.

**What Do These Findings Mean?:**

The results here suggest that this new haplotype matching method can provide a way to assess the risk of GVHD after HCT from unrelated donors, and in future could be considered as a technique to match donors and recipients.

**Additional Information.:**

Please access these Web sites via the online version of this summary at http://dx.doi.org/10.1371/journal.pmed.0040008.

• Medline Plus has a page of information on stem cell transplantation, including HCT

• The Anthony Nolan Trust holds one of the largest databases of unrelated donors in the world

• The National Cancer Institute has a page of questions and answers on HCT

• The Center for International Blood & Marrow Transplant Research describes outcomes research in transplantation

• The National Marrow Donor Program describes how HLA-typed unrelated donors are identified

• The World Marrow Donor Association is involved in facilitating stem cell donation across international boundaries

## Introduction

A hallmark of the human genome is its organization into segments or blocks of closely linked genetic variants that are inherited as haplotypes on the same DNA strand of a chromosome [1,2]. Genes that cause disease are located within haplotypes, and they can be identified by testing the association of disease with informative single nucleotide polymorphisms (SNPs) and other genomic markers [3]. Intense efforts are in progress to define the linkage of SNPs in genomic DNA, to specify the organization of SNPs into haplotype blocks, and to identify the SNPs that could serve as proxies for haplotype blocks (tagSNPs) [4,5]. This information provides powerful tools for mapping genes that cause disease [6]. When family studies are not available, haplotypes can be inferred with statistical methods [7,8]. Because haplotypes define not one, but many, physically linked markers, they provide important information regarding traits or diseases resulting from the additive effects of genetic variation that may alter disease susceptibility or severity and response to therapy [9–12].

Haplotypes of the human major histocompatibility complex (MHC) can be defined by using human leukocyte antigen (HLA) alleles as highly polymorphic markers. In human populations, multiple blocks of genetic variation in the MHC are strongly associated with each other as extended haplotypes [13–19]. Haplotypes that share the same HLA alleles may also share discrete segments or blocks of highly conserved sequences in strong positive LD with those HLA alleles [13,19–21]. HLA haplotypes serve as a model system for studies of disease association [22–26], but their biological implications have not been defined for solid organ and hematopoietic cell transplantation (HCT) [27,28]. In HCT, differences between polymorphic HLA antigens of the donor and recipient stimulate alloimmune reactions that cause graft-versus-host disease (GVHD) or graft rejection. HLA genotypically identical siblings are preferred as donors because the inheritance of identical MHC haplotypes by descent includes identity for all variation within the two parental MHC haplotypes. HLA-matched unrelated donors can be used for HCT when a related donor is not available [28–30]. The identification of HLA-matched unrelated donors is feasible because matching for two HLA loci will often determine matching for the third, a consequence of a phenomenon known as linkage disequilibrium (LD). HLA-allele matching has been used as a surrogate for MHC haplotype matching in an effort to reduce risks after unrelated HCT [28–30]. Nevertheless, GVHD remains a significant and potentially life-threatening complication after HLA-identical unrelated HCT [31].

Many of the more than 400 genes within the 7.6-million-base pair MHC region have immune-related functions, but the total number of transplantation antigens encoded within the MHC remains unknown. The dense clustering and strong LD of genes within the MHC could provide a framework for mapping genes that cause GVHD, if the extended MHC haplotypes of the unrelated donor and recipient could be defined. Current mapping strategies using selected tagSNPs to define MHC haplotype blocks that average 18,000 base pairs in length are not sufficient to define extended MHC haplotypes [14]. To overcome this limitation, we isolated DNA strands extending across 2 million base pairs of the MHC containing numerous haplotype blocks, and we then defined the *HLA-A, B, DR* haplotypes in recipients and their HLA-matched unrelated transplant donors [32]. The risks of post-transplant complications associated with haplotype mismatching were measured.

## Methods

### Study Participants

Patients were eligible (a) if they received a myeloablative conditioning regimen and T cell–replete HCT with bone marrow or growth-factor-mobilized blood cells from an *HLA-A, -B, -C, -DRB1, -DQB1* allele-identical unrelated donor for treatment of a blood disorder, and cyclosporine and methotrexate for post-grafting immunosuppression; (b) if lymphoblastoid cell lines were available from both the donor and recipient; and (c) if they were heterozygous at *HLA-B* and at *HLA-A* or *HLA-DRB1*. 246 patients met all three study criteria. 42 other patients were excluded because they were homozygous at two or all three loci (*n* = 22), homozygous at *HLA-B* (*n* = 5), or the *HLA-A* and *-DRB1* allele genotyping was ambiguous (*n* = 15); haplotyping of these pairs will be feasible in the future when the method is developed to include additional polymorphic loci. The institutional review board of the Fred Hutchinson Cancer Research Center approved the study, and informed consent was obtained from all patients.

### Donor HLA Selection Criteria

HLA-compatible unrelated donors were selected as previously described [33,34]. In the present study, retrospective sequencing was performed to confirm donor–recipient *HLA-A, -B, -C, -DRB1, -DQB1* allele identity and to determine *HLA-DPB1* alleles in 226 transplant pairs [33].

### Haplotyping to Determine Linkage of *HLA-A, -B,* and -*DRB1* Alleles

**Figure 1:**
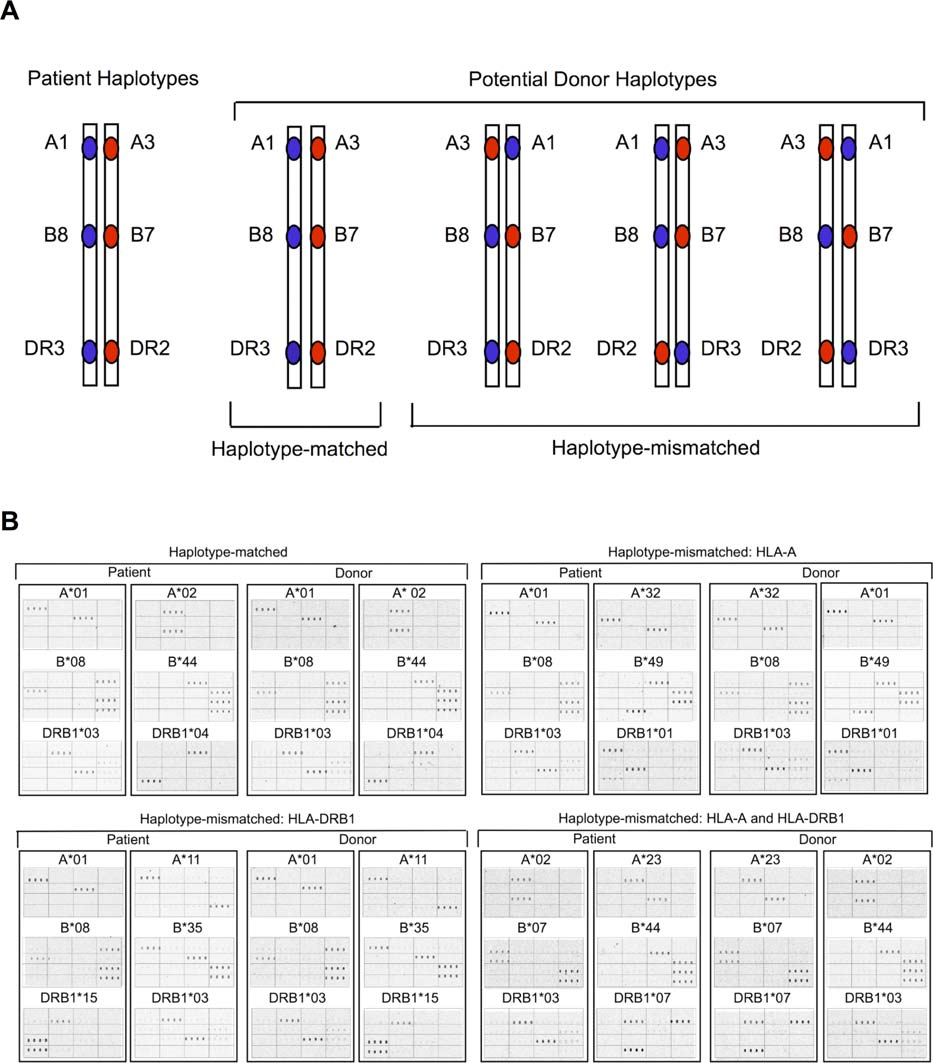
Long-Range Haplotyping of *HLA-A, -B,* and *-DRB1* in Unrelated Individuals (A) Schematic illustration of two HLA phenotypically identical individuals with the same or different linkages between *HLA-A, -B,* and *-DRB1* on the MHC haplotypes. (B) DNA microarray images of four unrelated donor–recipient pairs from the study population demonstrating MHC haplotype-matched (upper left), and MHC haplotype-mismatched (*HLA-A,* upper right; *HLA-DRB1,* lower left; *HLA-A* and -*DRB1,* lower right) relationships. The two haplotypes in each sample were separated by hybridizing genomic DNA to an array that was spotted with oligonucleotide probes, each specific for one of the two *HLA-B* alleles in the sample. After haplotype separation, the *HLA-A* and *HLA-DRB1* alleles carried on each haplotype were identified with the use of 57 *HLA-A* and 64 *HLA-DRB1* oligonucleotide probes as described [32]. Actual quadruplicate hybridization patterns for 16 of the probes illustrate how the two possible alleles at each locus could be distinguished from each other. Each column of panels in the figure shows the pattern of probe hybridization with one of the two MHC haplotypes from each sample. Allele assignments are indicated above each hybridization pattern. The *HLA-B* probe hybridization patterns validate the linkage of *HLA-B* alleles with *HLA-A* and -*DRB1* alleles. Sequences and specificity of probes can be found in [32].

We previously described a novel DNA microarray method to determine the physical linkage of *HLA-A, -B,* and *-DRB1* alleles when family study is not available [32]. Genomic DNA was extracted from lymphoblastoid cell lines under conditions that minimize shearing and then hybridized to arrays containing two oligonucleotide probes, each specific for the two *HLA-B* alleles in the sample. The linked *HLA-A* and *-DRB1* alleles on the captured *HLA-B* allele-specific strands were then genotyped by using oligonucleotide probes. Relative probe hybridization signal intensities were quantified with the aid of software dedicated to a fluorescence scanner and used to assign recipient and donor haplotypes. A pair was defined as MHC haplotype-matched if the *HLA-A, -B,* and *-DRB1* alleles were physically linked to each other on the captured haplotype; a pair was defined as MHC haplotype-mismatched if the *HLA-A, -B,* or *-DRB1* alleles were in any other rearrangement (Figure 1). The laboratory information was interpreted without knowledge of clinical outcomes and vice versa. In all cases, MHC haplotypes assigned by laboratory methods were concordant with those deduced from informative family studies [32]. In the present study, phase determination did not include consideration of *HLA-C, -DQB1,* or *-DPB1* loci. Analysis of these genes will be feasible with future modifications of the haplotyping method.

### Transplantation Procedure

Patients were prepared for transplantation with the use of myeloablative conditioning regimens [34] (Table 1). Pre-transplant disease risk was categorized as low-, intermediate-, or high-risk according to established criteria [34]. GVHD and recurrent malignancy were assessed as previously described [33]. In summary, conventional grading of acute GVHD incorporates the presence of the disease and its peak severity, which is related to the efficacy of treatment. Grade 0 indicates that the prophylactic immunosuppressive regimen administered after the transplant was sufficient to prevent clinical manifestations of GVHD. Grades I, II, III, and IV indicate that the immunosuppressive regimen was not sufficient to prevent clinical manifestations, and higher grades indicate increasing peak severity. Grade I GVHD indicates that manifestations were limited to the skin with less than 50% body surface involvement, and in most cases, the GVHD resolved spontaneously. Grade II GVHD indicates limited involvement of the gastrointestinal tract or liver, or more severe involvement of the skin, and in most cases, additional immunosuppressive treatment was sufficient to control disease. Grade III GVHD indicates more severe involvement of the gastrointestinal tract or liver, and in many cases, additional treatment did not readily control the disease. Grade IV GVHD indicates that GVHD was a major contributing cause of death. Transplant-related mortality and overall mortality are significantly higher among patients with grades III or IV acute GVHD compared to those with grades 0, I, or II GVHD. For these reasons, grades III–IV GVHD has been used as clinically meaningful endpoint in association analyses.

**Table 1:**
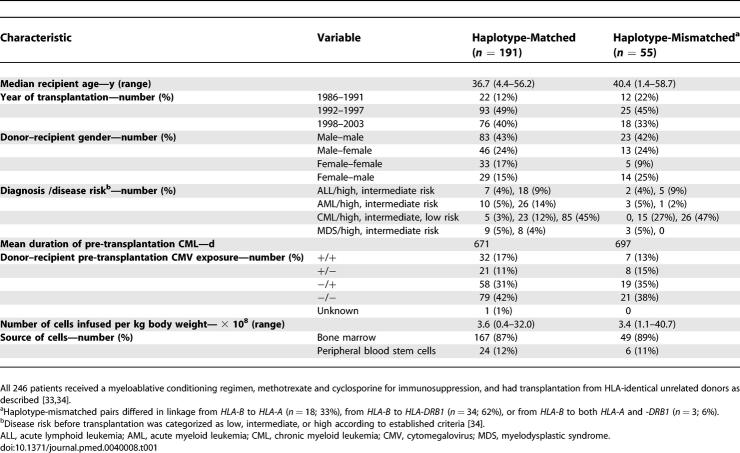
Characteristics of the Study Population

### Statistical Analyses

We used logistic regression to evaluate the association of MHC haplotype mismatching with grades III–IV acute GVHD. Cox regression was used to compare the hazards of recurrent malignancy, chronic GVHD, mortality, and transplant-related mortality. In addition to haplotype matching, all regression models contained variables for disease risk, patient age at transplantation, year of transplantation, patient/donor gender (female donor for male patient versus all other combinations), and presence of mismatching at *HLA-DPB1*. A test for interaction between number of common haplotypes and haplotype mismatching was performed by including the term “number × mismatch” in the regression model for GVHD, where “number” represents the number of common haplotypes modeled as a continuous linear variable, and “mismatch” is an indicator variable for haplotype mismatching. This amounted to testing if there was a linear increase (or decrease) in the odds ratios (ORs) of acute GVHD for haplotype mismatching compared to haplotype matching as the number of common haplotypes ranged from 0 to 2. All reported *p-*values are two-sided and were estimated from the Wald test, with no adjustments for multiple comparisons. Estimates of overall survival were calculated using the Kaplan–Meier method, and cumulative incidence estimates were used to summarize the probabilities of recurrent malignancy and transplant-related mortality. Death without recurrent malignancy was considered a competing risk for recurrent malignancy, and recurrent malignancy was considered a competing risk for transplant-related mortality.

## Results

### *HLA-A, -B, -DRB1* Linkage

A total of 246 *HLA-A, -B, -C, -DRB1, -DQB1* allele-matched transplants were evaluated in this study (Table 1). Of the 246 donor–recipient pairs, 191 (78%) were haplotype-matched and 55 (22%) were haplotype-mismatched. The haplotype-mismatched pairs differed in linkage from *HLA-B* to *HLA-A* (*n* = 18; 33%), from *HLA-B* to *HLA-DRB1* (*n* = 34; 62%) or from *HLA-B* to both *HLA-A* and -*DRB1* (*n* = 3; 6%). Recipients had a total of 262 different haplotypes, 197 of which were identified only once. Donors had a total of 299 different haplotypes, 239 of which were identified only once (Table S1).

### GVHD

**Figure 2:**
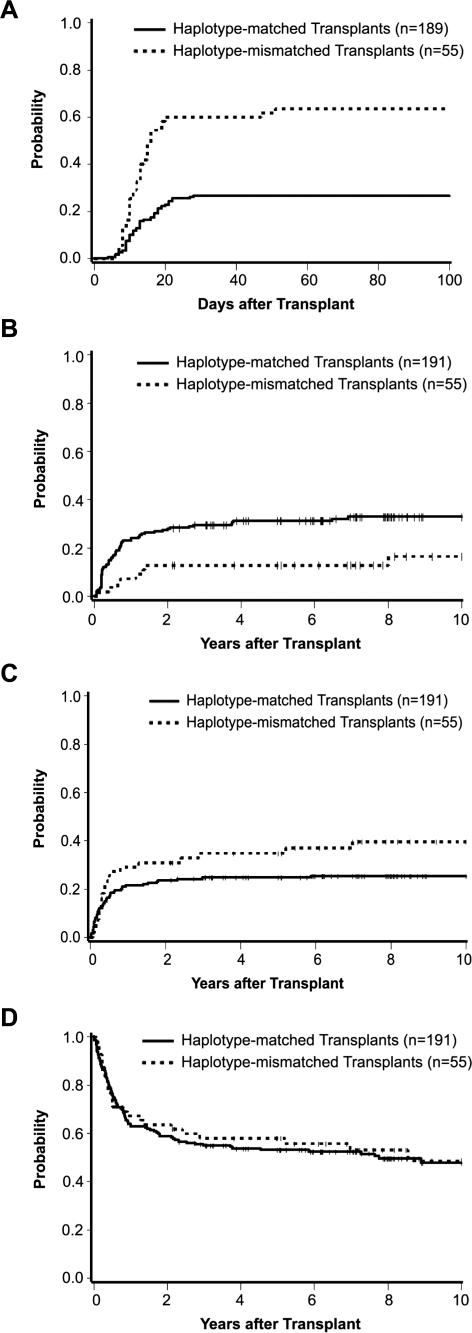
Clinical Outcome after Haplotype-Matched (Solid Line) and Haplotype-Mismatched (Broken Line) Unrelated Donor HCT All patients in the study were *HLA-A, B, C, DRB1, DQB1* allele matched with their donors. (A) Probability of grades III–IV acute GVHD. (B) Probability of recurrent malignancy. (C) Probability of transplant-related mortality. (D) Probability of survival. One patient in the mismatched group had recurrent malignancy at 14.4 y, and one patient in the mismatched group died without recurrent malignancy at 13.2 y. Seven mismatched patients are alive without recurrent malignancy from 11.9–14.1 y, and nine mismatched patients are alive from 11.0–14.5 y. Twenty-three patients in the matched group are alive without recurrent malignancy from 10.2–18.5 y, and 28 matched patients are alive from 10.2–18.5 y. Each of these patients is indicated as censored at 10 y in (B), (C), and (D).

Of the 246 patients in this study, two could not be evaluated for acute GVHD. One died on day 8 with multi-organ failure, and the other had recurrent malignancy without evidence of GVHD on day 19. Among the 244 patients who could be evaluated, the overall probability of grades III–IV acute GVHD was 34.8%. The probability of grades III–IV acute GVHD was 26.5% (2.6% grade IV) among haplotype-matched patients and 63.6% (3.6% grade IV) among haplotype-mismatched patients (Figure 2A). The percentages of haplotype-matched patients developing grade 0, I, II, III, and IV acute GVHD were 9.0, 4.0, 60.8, 23.8, and 2.6, respectively. Among haplotype-mismatched patients, the percentages developing grades 0, I, II, III, and IV were 3.6, 1.8, 30.9, 60.0, and 3.6, respectively. The probability of grades III–IV acute GVHD was 61% among haplotype-mismatched patients who differed in linkage from *HLA-B* to *HLA-A* and 62% among those who differed in linkage from *HLA-B* to *HLA-DRB1*.

The unadjusted OR for grades III–IV acute GVHD was 4.87 (95% confidence interval [CI], 2.57–9.20, *p* < 0.0001) for haplotype-mismatched patients compared to haplotype-matched patients. After adjusting for the factors described above, the OR of grades III–IV acute GVHD was 4.51 (95% CI, 2.34–8.70, *p* < 0.0001). The odds of GVHD conferred by haplotype mismatching were similar among patients matched at *HLA-DPB1* and patients mismatched at *HLA-DPB1* (ORs of 5.08 and 4.79, respectively).

The strong positive LD across the MHC that accounts for the association of certain *HLA-A, B,* and *DRB1* alleles on extended HLA haplotypes is not limited to common HLA alleles [13,18,20]. To determine whether the rate of haplotype matching is related to the presence of common haplotypes, we determined the haplotype match rate among patients with 0, 1, or 2 common haplotypes. Among the 246 patients, 229 (93%) were of Northern European descent and 17 (7%) were self-designated African, Asian, Hispanic, Native American, or other racial groups. Statistically inferred haplotypes occurring at a 0.2% or greater frequency in an unrelated donor population (http://www.nmdpresearch.org/HLA/em_haplotype_freq.html) were used to group the patients by the number of common haplotypes. Among the 229 patients of Northern European descent, 45 (19%) had no common haplotype, 130 (57%) had one, and 54 (24%) had two. For these three groups, the proportion of haplotype-matched donor–recipient pairs was 33/45 (73%), 102/130 (78%), and 42/54 (78%), respectively. Among the 17 non-Northern European patients, the proportion of haplotype-matched pairs was 3/6, 10/10, and 1/1, respectively.

Previous studies have described the presence of highly conserved regions between common HLA haplotypes that share the same HLA alleles [13,19,21,22]. Since haplotype mismatched pairs share certain HLA alleles and differ for others, the effect of haplotype mismatching could differ depending on the degree to which the mismatching haplotypes share conserved regions within the MHC. To address this question, we examined the association between haplotype mismatching and the probability of GVHD according to the number of common haplotypes. Among all donor–recipient pairs in the study, the magnitude of the effect of haplotype mismatching increased as the number of common haplotypes increased (Table 2), although the ORs were not statistically significantly different among the groups with 0, 1, or 2 common haplotypes (*p* = 0.20).

**Table 2:**
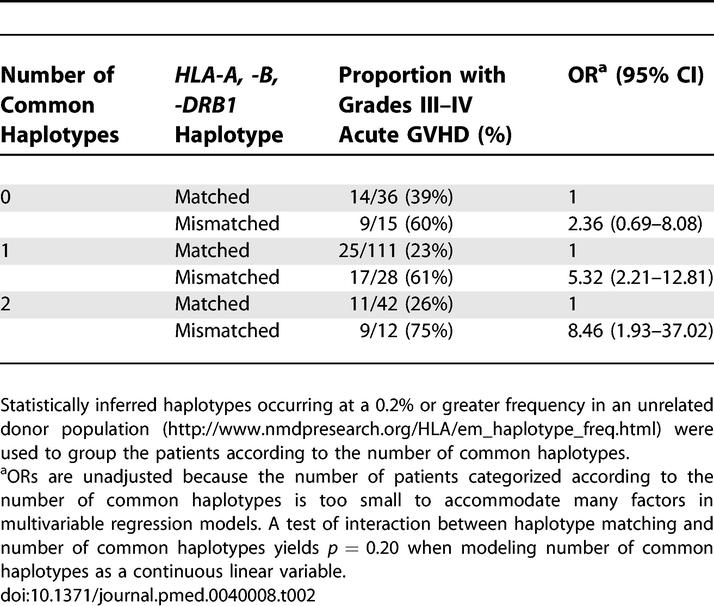
Probability of Grades III–IV Acute GVHD According to *HLA-A, -B, -DRB1* Haplotype Matching and Number of Common Haplotypes

A total of 42 donor–recipient pairs were homozygous at two or more loci, and could not be tested by our method. Of these 42 transplants, 12 (28.6%) developed grades III–IV acute GVHD, an incidence similar to that observed among the 189 haplotype-matched patients (26.5%).

Compared to haplotype-matched patients, haplotype-mismatched patients had an unadjusted hazard ratio (HR) of 1.03 for chronic GVHD (95% CI, 0.69–1.52, *p* = 0.90) and an adjusted HR of 1.05 (95% CI, 0.72–1.55, *p* = 0.79). 14% of haplotype-matched patients who developed grades III–IV acute GVHD died before day 100, compared to 11% among haplotype-mismatched patients.

### Recurrent Malignancy and Mortality

The increased risk of grades III–IV acute GVHD was accompanied by a statistically significantly decreased hazard of recurrent malignancy among haplotype-mismatched patients compared to haplotype-matched patients (HR 0.45; 95% CI, 0.22–0.92, *p* = 0.03) (Figure 2B; Table 3).

**Table 3:**
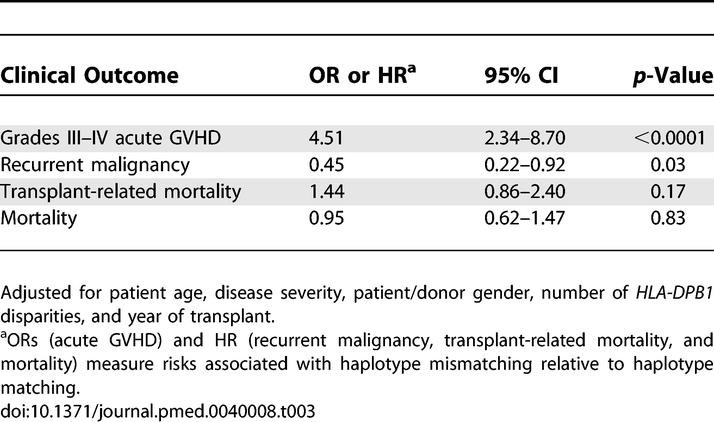
Adjusted Effect of *HLA-A, -B, -DRB1* Haplotype Mismatching on Risks of Grades III–IV Acute GVHD, Recurrent Malignancy, Transplant-Related Mortality, and Mortality after HCT from HLA-Identical Unrelated Donors

There was a suggestion of increased risk of transplant-related mortality with MHC haplotype mismatching, but this association was not statistically significant (HR 1.44; 95% CI, 0.86–2.40, *p* = 0.17) (Figure 2C; Table 3). Twenty-six of 45 (58%) haplotype-matched patients with grade III acute GVHD died (19 of the 26 deaths from transplant-related causes), and 15 of 33 (45%) haplotype-mismatched patients with grade III acute GVHD died (14 of the 15 deaths from transplant-related causes). Thirty-one of 50 (62%) haplotype-matched patients with grades III–IV acute GVHD died (24 deaths without prior disease recurrence), compared to 17 of 35 (49%) haplotype-mismatched patients (16 deaths without prior disease recurrence). The decrease in recurrent malignancy among haplotype-mismatched patients compared to haplotype-matched patients coupled with the increase in severe GVHD led to similar overall survival (Figure 2D) and adjusted hazards of mortality (HR 0.95; 95% CI, 0.62–1.47, *p* = 0.83).

## Discussion

In this study of HLA-matched transplant recipients and donors, haplotype mismatching was associated with a statistically significantly increased risk of severe acute GVHD. These results indicate that the HLA haplotype can serve as a proxy for GVHD risk. The advent of the polymerase chain reaction (PCR) assay in the 1980s ushered in the HLA molecular typing era and provides the basis for current criteria for donor selection in support of unrelated HCT (http://www.worldmarrow.org; http://www.nmdp.org). Although locus-by-locus matching of donor and recipient HLA alleles can reduce risks of graft failure and GVHD [28–30], HLA-identical transplant recipients still suffer from potentially life-threatening GVHD. Since HLA-matched unrelated donors and recipients are not identical by descent, we hypothesized that some donor–recipient pairs have identical MHC haplotypes of physically linked HLA alleles, while others have different haplotypes. Given the strong LD across the MHC [5,14,15,19,26], MHC haplotype matching may predict similarity for the more than 400 immune-related MHC-resident genes that could cause GVHD. Since it would not be feasible to assess each MHC polymorphism individually, we applied classical concepts of LD mapping to measure haplotype-associated risk [2,3,6].

Family studies are not available for unrelated transplant donors. Population statistics for individual haplotype inference may be inaccurate [35], and previously available laboratory techniques could not be used to define haplotypes across large distances within the MHC [24,36]. Application of our new method uncovered haplotype mismatching in 20% of HLA allele-matched unrelated pairs. The increment in GVHD risk associated with haplotype mismatching was at least comparable in magnitude, if not greater than the increment associated with a single HLA mismatch among related HCT recipients [37]. Since the patients and donors had identical *HLA-A, -B, -C, -DRB1,* and *-DQB1* alleles, GVHD could not have been caused by disparity for these classical HLA genes. Furthermore, the magnitude of the effect associated with haplotype mismatching was similar among patients matched and mismatched at *HLA-DPB1,* indicating that *HLA-DPB1* cannot explain the association of haplotype mismatching with GVHD. These results support the concept that the MHC harbors additional genes that encode transplantation antigens. Morbidity from GVHD after HCT might be lowered not only by matching the individual HLA alleles of the donor and recipient, but also by matching their extended MHC haplotypes.

The current study was not designed to examine any of the many genes outside the MHC that could cause GVHD [38]. Since these genes are not HLA linked, it is unlikely that they could have affected the association between MHC haplotype mismatching and risk of GVHD observed in the current study. Included among the many potential non-MHC variants that could contribute to GVHD are minor histocompatibility antigens such as those encoded by the Y chromosome when female donors are used for male recipients, or minor antigens associated with common HLA alleles such as HLA-A*0201 [39]. Notably, the frequency of HLA-A*0201 and the proportion of gender-mismatched pairs were similar in the haplotype-matched and haplotype-mismatched groups; furthermore, gender mismatching was included in the regression models. Therefore, any contribution to GVHD from these minor antigens would not be expected to influence the impact of haplotype mismatching on GVHD risk in the study population. Finally, *HLA-B* and *-C* determinants are known to serve as ligands for natural killer (NK) cell receptors [40]. Since all study pairs were *HLA-B* and *-C* allele matched, and since the distributions of NK ligands in the haplotype-matched and haplotype-mismatched groups were similar (unpublished data), NK ligands cannot explain our findings.

Mortality after HCT may be caused by transplant-related complications or by recurrent malignancy. The lack of a statistically significant association of MHC haplotype matching with overall survival was unexpected but may reflect the association of haplotype mismatching predominantly with clinically severe (grade III) GVHD rather than lethal (grade IV) GVHD. Even if the use of HLA-matched haplotype-matched unrelated donors does not improve overall survival, the effort to avoid or decrease the frequency of severe acute GVHD could provide clinical benefit. Donor–recipient haplotype matching may provide a novel strategy to decrease morbidity from GVHD and may be especially useful for patients who, because of medical reasons or advanced age, would not tolerate severe GVHD due to organ toxicity or prolonged immunosuppressive therapy. The patients in this study received T-replete unrelated donor grafts. Whether haplotype matching can further reduce GVHD risk after T cell–depleted transplantation remains to be determined. Further studies are warranted to define potential differences in the risk of mortality associated with grade III acute GVHD in haplotype-matched and haplotype-mismatched patients and to determine whether haplotype mismatching is associated with adverse outcomes after HCT with other preparative regimens, sources of stem cells, and immunosuppressive regimens.

Among haplotype-mismatched recipients, the increased risk of GVHD was offset by a lower risk of recurrent malignancy. Whether the lower risk of recurrent malignancy among haplotype-mismatched patients can be attributed to an increased risk of GVHD (through graft-versus-leukemia effects) or to causes that do not involve GVHD remains to be addressed in a larger transplant population [41]. At present, additional pharmacologic or immunotherapeutic strategies may be necessary in order to optimize overall transplant outcome if haplotype-matched donors are selected as a way of decreasing morbidity and mortality from GVHD in patients at high risk of recurrent malignancy after HCT.

The determinants that contribute to GVHD risk after haplotype-mismatched transplantation could be located anywhere within the gene-rich MHC, and the effects could arise from either disparity between the donor and recipient haplotype-linked variation or from the direct effects of the variation itself. Previous studies have delineated the sequence of several well-known common HLA haplotypes [13,16,19–22]. Haplotypes that share the same alleles at a given HLA locus also share highly conserved segments or blocks of sequences in strong LD with the HLA allele. Thus, the degree of similarity between haplotype-mismatched combinations could vary considerably, and certain regions within the MHC may contribute different levels of GVHD risk associated with haplotype mismatching. If common haplotypes are more highly conserved for undetected variation in certain regions of the MHC than others, then the effect of haplotype mismatching on GVHD among patients with common haplotypes might be greater than the effect among patients with no common haplotypes, as suggested by our current results. If future studies with larger numbers of patients validate this hypothesis, the results would suggest that efforts to identify haplotype-matched donors would provide the greatest level of protection against GVHD in the subpopulation of patients with two common haplotypes.

An important question is whether transplant outcomes can be improved by matching for selected regions of HLA haplotypes that contribute the highest risk of GVHD. Additional technology will be needed to allow all haplotype-associated MHC genes to be tested for their individual contributions to GVHD risk, and a very large cohort will be needed to answer this question. At present, the similarity of GVHD probabilities in patients whose haplotypes differed from *HLA-B* to *HLA-A* and from *HLA-B* to *HLA-DRB1* suggests that GVHD-risk genes are located both telomeric and centromeric to *HLA-B*. Furthermore, among the 191 haplotype-matched pairs, only four differed in linkage from *HLA-B* to *HLA-C*, and no cases differed in linkage from *HLA-DRB1* to *HLA-DQB1* (unpublished data). These results suggest that nearly all haplotype-matched pairs had identical five-locus haplotypes and that *HLA-C* and *HLA-DQB1* provide additional markers for narrowing the potential regions of interest. Candidates include any gene-encoding polymorphic determinants that can function as transplantation antigens [14,16,17,19,42]. If only a few genes contribute to the increased risk of GVHD in haplotype-mismatched patients, refinement of our haplotyping method to include informative MHC class I, II, and III markers could be used to narrow the boundaries of GVHD-risk regions, allowing smaller regions to be examined at a higher level of genomic discrimination [14,19,26,42].

Specific regions of mismatching between the donor and recipient MHC haplotypes might also have different effects on the risk of recurrent malignancy. In the current study, the limited numbers of class I and class II haplotype-mismatched patients precluded any informative analysis of the association of recurrent malignancy with class I versus class II haplotype mismatching. Information from future studies showing differences in the risk of recurrent malignancy associated with haplotype mismatching for specific MHC regions could facilitate development of an immunogenetic approach toward decreasing the risk of acute GVHD while preserving graft-versus-leukemia effects.

Regardless of the number, nature, and location of GVHD-risk genes within the MHC, the results of the current study demonstrate that the three-locus *HLA-A, -B, -DRB1* haplotype can serve as a proxy for GVHD risk. The ease of our haplotyping technique focused on only three genetic markers could provide clinicians with a simple tool for GVHD risk assessment and a means to decrease GVHD-associated morbidity after unrelated HCT. In the future, more refined mapping of GVHD-risk determinants will provide important information as to whether matching for certain regions of haplotypes will be clinically important.

Currently, patients with common HLA alleles in strong LD have the highest chances of identifying HLA allele-matched donors. We were interested in addressing whether the presence of common haplotypes in a recipient could be used as a predictor of haplotype matching, since this information has practical implications in the search for unrelated donors. Among the entire study population, the rate of haplotype matching was 78% for patients with two common haplotypes, 80% for those with one, and 71% for those with none (Table 2). Although the number of study participants was limited, these data suggest that allele frequency is only one parameter of successful donor haplotype matching.

Strong positive LD between low-frequency alleles increases the chance that a haplotype-matched donor might be identified for a given patient. For the 55 haplotype-mismatched patients in our study, a repeated search of the Bone Marrow Donors Worldwide database (http://www.bmdw.org) identified between four and 1,777 (median: 65) potential *HLA-A, -B, -DRB1*-matched donors per patient. For the 45 patients of Northern European descent with two uncommon haplotypes, a repeated search identified up to 321 (median: 14) potential *HLA-A, -B, -DRB1*-matched donors per patient. One patient had a single donor, and only one patient had no other potential donors. These observations suggest that most five-locus-matched Northern European patients have several potential donors who could be prospectively haplotyped. Development of strategies to maximize the identification of potential haplotype-matched donors among the 10 million volunteer donors worldwide (http://www.bmdw.org; http://www.nmdp.org) and to direct donor recruitment efforts in a way that provides optimal size and composition of registries ([43,44]; http://www.worldmarrow.org) will be important research questions for the future.

Haplotypes have very high utility in hypothesis-driven and exploratory gene mapping. Our method for long-range haplotype definition could be used to assess the clinical importance of haplotypes in solid organ transplantation [27] and to discover genes that predispose to autoimmunity, infection, and cancer [23,25,45–47]. This method offers the potential for disease-association mapping without the need for individual tagSNP selection. Previous studies have been confounded by strong LD, which makes it difficult to determine whether disease susceptibility is conferred by the marker gene itself or by another gene in LD with the marker gene. Our haplotyping method will not surmount the problem of strongly linked genes, but it could be used to clarify physical linkages that differ between cases and controls and permit the measurement of independent effects. The ability to isolate high-quality intact genomic DNA for extended haplotyping provides an approach for screening causative variants even if the full extent of human haplotype diversity and structure are yet to be uncovered.

## Supporting Information

Alternative Language Abstract S1 Translation of the Abstract into German by H. J. Deeg (21 KB DOC)

Alternative Language Abstract S2 Translation of the Abstract into French by L. Loubière (25 KB DOC)

Alternative Language Abstract S3 Translation of the Abstract into Spanish by J. C. Pizarro (25 KB DOC)

Alternative Language Abstract S4 Translation of the Abstract into Chinese by H. Wang (21 KB DOC)

Alternative Language Abstract S5 Translation of the Abstract into Japanese by N. Fujii Found at doi:10.1371/journal.pmed.0040008.sd005 27 KB DOC).

Table S1 *HLA-A, -B, -DRB1* Haplotypes in the Study Population (396 KB DOC)

### Accession Numbers

The Entrez database (http://www.ncbi.nlm.nih.gov/entrez/query.fcgi?DB=pubmed) gene identifiers for *HLA-A*, *-B, -C, -DRB1, -DQB1,* and *-DPB1* are 3105, 3106, 3107, 3123, 3119, and 3115, respectively.

## Abbreviations

CI: confidence interval
GVHD: graft-versus-host disease
HCT: hematopoietic cell transplantation
HLA: human leukocyte antigen
HR: hazard ratio
LD: linkage disequilibrium
MHC: major histocompatibility complex
NK: natural killer
OR: odds ratio
SNP: single nucleotide polymorphism
