# Supplementary material for: MHC Haplotype Matching for Unrelated Hematopoietic Cell Transplantation
Source: PLoS Med. 2007 Jan 30;4(1):e8. doi: 10.1371/journal.pmed.0040008 (PMC1796628; doi:10.1371/journal.pmed.0040008)
Supplement: Alternative Language Abstract S1 — (21 KB DOC) [file pmed.0040008.sd001.doc]

**Grundlagen**

Das wichtigste Kriterium zur Auswahl von unverwandten Spendern fuer die Transplantation von haematopoietischen Zellen (HZT) ist die Identitaet fuer Allele aller HLA Loci innerhalb des Histokompatibilitaetskomplexes (MHC). Die Transplantat-gegen-Wirt Erkrankung (Graft-versus-Host Disease [GVHD]) ist aber nach wie vor eine schwere und potentiell toedliche Komplikation, selbst mit HLA identischen Spendern. Der MHC umfasst mehr als 400 Gene, aber die Gesamtzahl der fuer die Transplantation relevanten Gene ist nicht bekannt. Solche Gene koennten bestimmt werden durch Linkagedisequilibrium Methoden sofern man den ‚extended haplotype’ von Spender und Empfaenger charakterisieren kann.

**Methodik und Ergebnisse**

Wir isolierten DNA Molekuele, die sich ueber zwei Millionen Basenpaare des MHC erstreckten und bestimmten die Linkage von HLA-A, -B und DRB1 Allelen in 246 HZT Patienten und ihren HLA-A, -B, -C,-DRB1 und DQB1 allel-identischen unverwandten Spendern. Nichtidentitaet fuer den MHC zeigte eine statistisch significante Korrelation mit erhoehtem Risiko fuer schwere GVHD (Odds ratio 4.51; 95% Konfidenzintervall [KI] 2.34 – 8.70; p<0.0001) und mit geringerem Rezidivrisiko (hazard ratio 0.45; 95% KI 0.22-0.92; p=0.03).

**Schlussfolgerungen**

Der MHC enthaelt Gene fuer bisher nicht charakterisierte Transplantationsantigene. Der Drei-Loci Haplotyp HLA-A, -B, -DRB1 bestimmt nur annaeherungsweise das GVHD Risiko in HLA-identischen Transplantatempfaengern . Unsere „phasing“ Methode erlaubt uns neue MHC-assoziierte Transplantationsantigene zu bestimmen und dadurch die mit GVHD verbundene Morbiditaet nach unverwandter Transplantation zu verringern.
