# Supplementary material for: MHC Haplotype Matching for Unrelated Hematopoietic Cell Transplantation
Source: PLoS Med. 2007 Jan 30;4(1):e8. doi: 10.1371/journal.pmed.0040008 (PMC1796628; doi:10.1371/journal.pmed.0040008)
Supplement: Alternative Language Abstract S2 — (25 KB DOC) [file pmed.0040008.sd002.doc]

**Contexte ou Introduction**

Les critères actuels de sélection des donneurs non apparentés pour transplantation de cellules souches hématopoïétiques (CSH) incluent l’appariement des allèles de chaque locus du HLA au sein du complexe majeur d’histocompatibilité (CMH). Cependant, la maladie du greffon contre l’hôte (GVHD) reste une complication majeure et potentiellement mortelle même dans le cas de transplants provenant de donneurs non apparentés HLA identiques. Le CMH contient plus de 400 gènes, mais le nombre total d’antigènes de transplantation reste inconnu. Si les haplotypes au sens large du CMH du donneur non apparenté et du receveur pouvaient être définis alors les gènes qui influencent le résultat de la transplantation pourraient être identifiés par l’utilisation d’approches basées sur la cartographie des déséquilibres de liaison (DL).

**Méthodes et Résultats**

Nous avons isolé des fragments d’ADN couvrant les 2 millions de paires de bases du CMH afin de déterminer la liaison physique des allèles HLA-A, -B et -DRB1 de 246 receveurs de transplant et leurs donneurs appariés pour les allèles HLA-A, -B, -C, -DRB1, et -DQB1. Le mésappariement des haplotypes CMH était associé à une augmentation statistiquement significative du risque de GVHD sévère et aiguë (odds ratio 4.51; intervalle de confiance [IC] à 95%, 2.34-8.70, p < 0.0001) et à un faible risque de récurrence de la maladie (risque relatif 0.45 ; IC à 95%, 0.22-0.92, p = 0.03).

**Conclusions**

Le CMH héberge des gènes codant pour des antigènes de transplantation encore non identifiés. L’haplotype constitué des trois locus HLA-A, -B, -DRB1 sert de déterminant du risque de GVHD parmi les receveurs de transplants HLA identiques. La méthode déterminant la position en cis ou trans des allèles HLA fournis une approche pour la cartographie de nouveaux facteurs associés au CMH contrôlant le succès de la transplantation, et un moyen de réduire la morbidité liée à la GVHD après HCT de donneur non apparentés.
