# Supplementary material for: MHC Haplotype Matching for Unrelated Hematopoietic Cell Transplantation
Source: PLoS Med. 2007 Jan 30;4(1):e8. doi: 10.1371/journal.pmed.0040008 (PMC1796628; doi:10.1371/journal.pmed.0040008)
Supplement: Alternative Language Abstract S3 — (25 KB DOC) [file pmed.0040008.sd003.doc]

**Antecedentes**

Los criterios actuales de selección para donantes no relacionados para el transplante de medula ósea (HCT sigla en ingles) incluyen el apareamiento de los alelos HLA dentro del complejo de mayor histocompatibilidad (MHC sigla en ingles). Sin embargo, la enfermedad aguda de injerto versus huésped (GVHD sigla en inglés) continua siendo un riesgo mortal, aun en casos de emparejamiento perfecto de los alelos HLA para donantes no relacionados del HCT. El MHC contiene mas de 400 genes, pero el numero total de antígenos de transplante es aun desconocido. Los genes que influyen sobre el resultado de un transplante pueden ser identificados utilizando una aproximación de mapeo utilizando el desequilibrio de ligamiento (LD sigla en ingles), si los haplotipos extendidos del MHC pueden ser definidos para el donante no relacionado y su receptor.

**Métodos y resultados**

Se aisló el ADN extendiéndose a través de dos millones de pares de bases del MHC para determinar el ligamiento físico entre los alelos de los genes HLA-A, -B y –DRB1 proveniente de 246 células hematopoyéticas de los recetores del transplante y los genes HLA-A, -B, -C, -DBR1 y -DQB1 de sus donantes no relacionados. Las incompatibilidades de los haplotipos fueron asociadas con un aumento estadísticamente significativo de la GVHD (*razón de probabilidades* 4.51; intervalo de confianza [IC] 2.34 - 8.70, p<0.0001), al mismo tiempo una menor probabilidad de reincidencia (riesgo relativo 0.45; 95% IC, 0.22 - 0.92, p=0.03).

**Conclusiones**

El MHC incluye genes que codifican antígenos de transplante no identificados. El haplotipo proveniente de los tres locus HLA-A, -B, -DBR1 sirve de pronostico de riesgo de GVHD para transplantes de recipientes idénticos. El *método de fases* (phasing method) provee una novedosa aproximación para el mapeo de determinación de vínculos al MHC, además de reducir la mortalidad relacionada al GVHD después de una HCT de donadores no relacionados.
