# Supplementary material for: MHC Haplotype Matching for Unrelated Hematopoietic Cell Transplantation
Source: PLoS Med. 2007 Jan 30;4(1):e8. doi: 10.1371/journal.pmed.0040008 (PMC1796628; doi:10.1371/journal.pmed.0040008)
Supplement: Alternative Language Abstract S4 — (21 KB DOC) [file pmed.0040008.sd004.doc]

**研究背景**

目前的造血细胞移植（hematopoietic cell transplantation，HCT）中，筛选非血缘性供体的标准包括分析对位于主要组织相容性复合体（major histocomapatibility complex，MHC）的每种白细胞座位（HLA座位）的等位基因的匹配结果。然而，即使HLA座位识别非血缘性造血细胞，移植物抗宿主疾病(graft-versus-host-disease，GVHD)仍然是一种显著而潜在的威胁生命的并发病。主要组织相容性复合体内含有400多种基因，然而移植抗原的总数是一未知数。如果能够确定非血缘性供体和受体的相引的MHC单模式，则可以使用连锁不平衡基因匹配方法鉴别影响造血细胞移植结果的基因。

**方法和发现**

从MHC中，我们分离提取包含2百万个碱基对的DNA双链。该DNA双链用于测定246位造血细胞移植受体的白细胞座位A, B和DRB1的物理连锁图，以及等位基因匹配的非血缘性供体的白细胞座位A, B, C, DRB1和DQB1的物理连锁图。通过对结果进行统计分析后，发现主要组织相容性复合体单模式的错配与显著性地增加严重的急性GVHD的危险率和降低疾病复发的危险率有关，其结果为：

显著性地增加严重的急性GVHD的危险率：

（优势比率： 4.51；95%概率的置信区间：2.34-8.70；*p*<0.0001）。

降低疾病复发的危险率：

（危险比率： 0.45；95%概率的置信区间：0.22-0.92；*p*<0.03）。

**结论**

位于MHC的基因编码未知移植抗原。在对白细胞座位识别的造血细胞移植受体中，白细胞座位HLA-A，HLA-B，和HLA-DRB1单模式可以作为评估GVHD危险率的替代数据。本研究提供了一种匹配新的与主要组织相容性复合体关联的移植决定簇，并可以用于降低非血缘性供体的HCT术后的与GVHD有关的疾病的发病率。
