# Supplementary material for: MHC Haplotype Matching for Unrelated Hematopoietic Cell Transplantation
Source: PLoS Med. 2007 Jan 30;4(1):e8. doi: 10.1371/journal.pmed.0040008 (PMC1796628; doi:10.1371/journal.pmed.0040008)
Supplement: Alternative Language Abstract S5 — Found at doi:10.1371/journal.pmed.0040008.sd005 27 KB DOC). [file pmed.0040008.sd005.doc]

**背景**
現在、非血縁者間造血細胞移植でのドナーは、主要組織適合複合体に含まれるそれぞれのHLAアレルの一致度に基づいて選択されている。しかしながら、非血縁者間造血細胞移植においては、HLAが一致していても、移植片対宿主病は依然として重要でしばしば生命を脅かす合併症である。主要組織適合複合体には400以上の遺伝子が含まれるが、そのうちのいくつが移植成績に影響を与える抗原であるかは分かっていない。非血縁者間移植におけるドナーと患者の主要組織適合複合体のハプロタイプが詳細に定義されれば、連鎖不平衡地図の手法を用いて、移植成績に影響を与える遺伝子を特定できる可能性がある。

**方法と結果**
HLA-A、-B、-DRB1アレルの連鎖解析を行うために、HLA-A、-B、-C、-DRB1、-DQB1が一致している非血縁造血細胞移植において、246人の造血細胞移植患者とドナーから、主要組織適合複合体を含む200万塩基対のDNAを分離した。主要組織適合複合体ハプロタイプが一致していない移植においては、重症急性移植片対宿主病が起こる確率が高く（オッズ比；4.51、95%信頼区間；2.34-8.70、p<0.0001）、原病の再発率は低かった（ハザード比；0.45、95%信頼区間；0.22-0.92、p = 0.03）。

**結論**
主要組織適合複合体には、未知の移植抗原をコードする遺伝子が含まれている。HLAが一致している非血縁者間移植においては、HLA-A、-B、-DRB1の３遺伝子座のハプロタイプによって移植片対宿主病の危険性を予測することができる。今回の研究を通して主要組織適合複合体内に新たな移植抗原を同定することによって、非血縁者間造血細胞移植にお
ける移植片対宿主病に関連した合併症を減らせる可能性がある。
