# Supplementary material for: MHC Haplotype Matching for Unrelated Hematopoietic Cell Transplantation
Source: PLoS Med. 2007 Jan 30;4(1):e8. doi: 10.1371/journal.pmed.0040008 (PMC1796628; doi:10.1371/journal.pmed.0040008)
Supplement: Table S1 — (396 KB DOC) [file pmed.0040008.st001.doc]

| **Table S1.** *HLA-A, -B, -DRB1* Haplotypes in the Study Population | | | |
| --- | --- | --- | --- |
|  |  |  |  |
| *HLA-A, -B, -DRB1* Haplotype | Number of Haplotypes  in Recipients (n=492) | Number of Haplotypes  in Donors (n=492) | Total Number of Haplotypes  in Recipients and Donors (n=984) |
| 0101- 0801- 0301 | 57 | 54 | 111 |
| 0301- 0702- 1501 | 26 | 23 | 49 |
| 0201- 4402- 0401 | 18 | 16 | 34 |
| 0201- 1501- 0401 | 11 | 9 | 20 |
| 2902- 4403- 07 | 11 | 9 | 20 |
| 0301- 3501- 0101 | 9 | 8 | 17 |
| 0201- 0702- 1501 | 8 | 9 | 17 |
| 2601- 3801- 0402 | 7 | 5 | 12 |
| 0201- 0801- 0301 | 6 | 5 | 11 |
| 3002- 1801- 0301 | 6 | 4 | 10 |
| 0201- 1501- 1301 | 4 | 4 | 8 |
| 0101- 5701- 07 | 4 | 3 | 7 |
| 0201- 4403- 07 | 4 | 3 | 7 |
| 3301- 1402- 0102 | 4 | 3 | 7 |
| 0101- 5701- 1305 | 4 | 2 | 6 |
| 2301- 4403- 07 | 4 | 2 | 6 |
| 0201- 1302- 07 | 3 | 3 | 5 |
| 0201- 5701- 07 | 3 | 3 | 6 |
| 0201- 2705- 0101 | 3 | 2 | 5 |
| 0201- 4001- 0401 | 3 | 2 | 5 |
| 0201- 4001- 1302 | 3 | 2 | 5 |
| 0201- 4402- 1301 | 3 | 2 | 5 |
| 0301- 4701- 07 | 3 | 2 | 5 |
| 2402- 3502- 1104 | 3 | 2 | 5 |
| 2501- 1801- 1501 | 3 | 2 | 5 |
| 3001- 1302- 07 | 3 | 2 | 3 |
| 3201- 1401- 07 | 3 | 2 | 5 |
| 6801- 4001- 1302 | 3 | 2 | 5 |
| 0101- 0702- 1501 | 3 | 1 | 4 |
| 0101- 3701- 10 | 3 | 1 | 4 |
| 0201- 1501- 0101 | 2 | 3 | 5 |
| 0201- 1801- 0301 | 2 | 3 | 5 |
| 0201- 4402- 1104 | 2 | 3 | 5 |
| 2402- 0702- 1501 | 2 | 3 | 5 |
| 0101- 4402- 0402 | 2 | 2 | 4 |
| 0201- 3503- 1201 | 2 | 2 | 4 |
| 0201- 4001- 0801 | 2 | 2 | 4 |
| 0201- 4402- 0101 | 2 | 2 | 4 |
| 0201- 4402- 0301 | 2 | 2 | 4 |
| 0201- 4402- 07 | 2 | 2 | 4 |
| 0301- 0702- 0101 | 2 | 2 | 4 |
| 0301- 1402- 0102 | 2 | 2 | 4 |
| 0301- 2705- 0101 | 2 | 2 | 4 |
| 0301- 5101- 07 | 2 | 2 | 4 |
| 1101- 3501- 0101 | 2 | 2 | 4 |
| 2402- 1801- 0301 | 2 | 2 | 4 |
| 2402- 2705- 0101 | 2 | 2 | 4 |
| 2402- 5501- 1302 | 2 | 2 | 4 |
| 2902- 4402- 07 | 2 | 2 | 4 |
| 3301- 1402- 0301 | 2 | 2 | 4 |
| 6801- 1801- 0301 | 2 | 2 | 4 |
| 0101- 0801- 0101 | 2 | 1 | 3 |
| 0201- 0702- 0404 | 2 | 1 | 3 |
| 0201- 0702- 1301 | 2 | 1 | 3 |
| 0201- 4001- 0404 | 2 | 1 | 3 |
| 0201- 4402- 1201 | 2 | 1 | 3 |
| 0201- 5101- 0801 | 2 | 1 | 3 |
| 0301- 1402- 1302 | 2 | 1 | 3 |
| 0301- 1501- 0401 | 2 | 1 | 3 |
| 0302- 3901- 1101 | 2 | 1 | 3 |
| 1101- 2705- 0101 | 2 | 1 | 3 |
| 1101- 5501- 1401 | 2 | 1 | 3 |
| 1101- 5601- 0101 | 2 | 1 | 3 |
| 1101- 0702- 1501 | 2 |  | 2 |
| 3101- 4001- 0404 | 2 |  | 2 |
| 0101- 4001- 0404 | 1 | 2 | 3 |
| 0201- 0702- 0401 | 1 | 2 | 3 |
| 0201- 2705- 0404 | 1 | 2 | 3 |
| 0201- 4001- 0301 | 1 | 2 | 3 |
| 0201- 4402- 0404 | 1 | 2 | 3 |
| 0201- 4402- 1501 | 1 | 2 | 3 |
| 0201- 4403- 1501 | 1 | 2 | 3 |
| 0301- 4001- 1302 | 1 | 2 | 3 |
| 1101- 3501- 1501 | 1 | 2 | 3 |
| 2601- 1401- 07 | 1 | 2 | 3 |
| 6801- 0702- 1501 | 1 | 2 | 3 |
| 0101- 0801- 0401 | 1 | 1 | 2 |
| 0101- 0801- 1101 | 1 | 1 | 2 |
| 0101- 0801- 1103 | 1 | 1 | 2 |
| 0101- 0801- 1501 | 1 | 1 | 2 |
| 0101- 1401- 07 | 1 | 1 | 2 |
| 0101- 1501- 1301 | 1 | 1 | 2 |
| 0101- 1801- 0301 | 1 | 1 | 2 |
| 0101- 3701- 1302 | 1 | 1 | 2 |
| 0101- 4001- 1301 | 1 | 1 | 2 |
| 0101- 4402- 1501 | 1 | 1 | 2 |
| 0101- 4403- 07 | 1 | 1 | 2 |
| 0201- 0702- 0301 | 1 | 1 | 2 |
| 0201- 0702- 1101 | 1 | 1 | 2 |
| 0201- 1302- 1301 | 1 | 1 | 2 |
| 0201- 1501- 09 | 1 | 1 | 2 |
| 0201- 1501- 1303 | 1 | 1 | 2 |
| 0201- 1518- 1301 | 1 | 1 | 2 |
| 0201- 1801- 1104 | 1 | 1 | 2 |
| 0201- 1801- 1501 | 1 | 1 | 2 |
| 0201- 2705- 0301 | 1 | 1 | 2 |
| 0201- 3701- 0401 | 1 | 1 | 2 |
| 0201- 3701- 0801 | 1 | 1 | 2 |
| 0201- 4001- 09 | 1 | 1 | 2 |
| 0201- 4001- 1301 | 1 | 1 | 2 |
| 0201- 4402- 0803 | 1 | 1 | 2 |
| 0201- 4402- 1101 | 1 | 1 | 2 |
| 0201- 4402- 1302 | 1 | 1 | 2 |
| 0201- 4402- 1401 | 1 | 1 | 2 |
| 0201- 4403- 0101 | 1 | 1 | 2 |
| 0201- 4405- 07 | 1 | 1 | 2 |
| 0201- 5001- 1301 | 1 | 1 | 2 |
| 0201- 5101- 0408 | 1 | 1 | 2 |
| 0201- 5201- 1502 | 1 | 1 | 2 |
| 0201- 5501- 1501 | 1 | 1 | 2 |
| 0201- 7301- 0405 | 1 | 1 | 2 |
| 0202- 4901- 1501 | 1 | 1 | 2 |
| 0205- 4101- 07 | 1 | 1 | 2 |
| 0301- 0702- 0102 | 1 | 1 | 2 |
| 0301- 0702- 0407 | 1 | 1 | 2 |
| 0301- 0702- 0801 | 1 | 1 | 2 |
| 0301- 0702- 1101 | 1 | 1 | 2 |
| 0301- 0702- 1401 | 1 | 1 | 2 |
| 0301- 1402- 1501 | 1 | 1 | 2 |
| 0301- 1501- 0101 | 1 | 1 | 2 |
| 0301- 1501- 1301 | 1 | 1 | 2 |
| 0301- 1517- 1302 | 1 | 1 | 2 |
| 0301- 1801- 0401 | 1 | 1 | 2 |
| 0301- 1801- 1401 | 1 | 1 | 2 |
| 0301- 2702- 1601 | 1 | 1 | 2 |
| 0301- 2705- 0401 | 1 | 1 | 2 |
| 0301- 2705- 07 | 1 | 1 | 2 |
| 0301- 3503- 1501 | 1 | 1 | 2 |
| 0301- 4001- 0404 | 1 | 1 | 2 |
| 0301- 4101- 0404 | 1 | 1 | 2 |
| 0301- 4402- 1601 | 1 | 1 | 2 |
| 0301- 4701- 1301 | 1 | 1 | 2 |
| 0301- 5101- 1101 | 1 | 1 | 2 |
| 0301- 5501- 07 | 1 | 1 | 2 |
| 0301- 5501- 1601 | 1 | 1 | 2 |
| 0301- 5601- 0101 | 1 | 1 | 2 |
| 0301- 5601- 0801 | 1 | 1 | 2 |
| 0301- 5701- 0402 | 1 | 1 | 2 |
| 1101- 0801- 0301 | 1 | 1 | 2 |
| 1101- 1801- 07 | 1 | 1 | 2 |
| 1101- 4001- 1302 | 1 | 1 | 2 |
| 1101- 4402- 0401 | 1 | 1 | 2 |
| 1101- 5101- 1401 | 1 | 1 | 2 |
| 1101- 5501- 0101 | 1 | 1 | 2 |
| 2301- 0702- 1501 | 1 | 1 | 2 |
| 2301- 4901- 1101 | 1 | 1 | 2 |
| 2301- 5001- 0301 | 1 | 1 | 2 |
| 2402- 0702- 0101 | 1 | 1 | 2 |
| 2402- 0801- 0301 | 1 | 1 | 2 |
| 2402- 0802- 0801 | 1 | 1 | 2 |
| 2402- 1301- 1202 | 1 | 1 | 2 |
| 2402- 1501- 1103 | 1 | 1 | 2 |
| 2402- 1801- 0101 | 1 | 1 | 2 |
| 2402- 1801- 1104 | 1 | 1 | 2 |
| 2402- 2702- 1601 | 1 | 1 | 2 |
| 2402- 2705- 1401 | 1 | 1 | 2 |
| 2402- 3801- 1301 | 1 | 1 | 2 |
| 2402- 3801- 1401 | 1 | 1 | 2 |
| 2402- 3901- 04 | 1 | 1 | 2 |
| 2402- 3906- 1301 | 1 | 1 | 2 |
| 2402- 4001- 1302 | 1 | 1 | 2 |
| 2402- 4001- 1602 | 1 | 1 | 2 |
| 2402- 4402- 1104 | 1 | 1 | 2 |
| 2402- 4501- 10 | 1 | 1 | 2 |
| 2402- 4601- 09 | 1 | 1 | 2 |
| 2402- 4801- 1101 | 1 | 1 | 2 |
| 2402- 5101- 0301 | 1 | 1 | 2 |
| 2402- 5501- 1401 | 1 | 1 | 2 |
| 2402- 5601- 0101 | 1 | 1 | 2 |
| 2501- 0801- 0301 | 1 | 1 | 2 |
| 2501- 1501- 0101 | 1 | 1 | 2 |
| 2501- 1801- 07 | 1 | 1 | 2 |
| 2501- 1801- 1401 | 1 | 1 | 2 |
| 2501- 4405- 0101 | 1 | 1 | 2 |
| 2501- 5801- 1303 | 1 | 1 | 2 |
| 2601- 0702- 1501 | 1 | 1 | 2 |
| 2601- 1401- 0401 | 1 | 1 | 2 |
| 2601- 1801- 1301 | 1 | 1 | 2 |
| 2601- 3501- 0402 | 1 | 1 | 2 |
| 2601- 3801- 0102 | 1 | 1 | 2 |
| 2601- 3801- 07 | 1 | 1 | 2 |
| 2601- 4002- 09 | 1 | 1 | 2 |
| 2601- 4901- 07 | 1 | 1 | 2 |
| 2601- 5101- 1101 | 1 | 1 | 2 |
| 2601- 5501- 0101 | 1 | 1 | 2 |
| 2601- 5701- 07 | 1 | 1 | 2 |
| 2901- 0705- 10 | 1 | 1 | 2 |
| 2902- 0702- 1501 | 1 | 1 | 2 |
| 2902- 1302- 07 | 1 | 1 | 2 |
| 2902- 1402- 0303 | 1 | 1 | 2 |
| 2902- 1402- 07 | 1 | 1 | 2 |
| 2902- 4403- 0404 | 1 | 1 | 2 |
| 2902- 4403- 1501 | 1 | 1 | 2 |
| 3001- 1302- 0301 | 1 | 1 | 2 |
| 3001- 1302- 1101 | 1 | 1 | 2 |
| 3001- 1302- 1601 | 1 | 1 | 2 |
| 3001- 5301- 1101 | 1 | 1 | 2 |
| 3004- 5801- 1502 | 1 | 1 | 2 |
| 3101- 3802- 1101 | 1 | 1 | 2 |
| 3101- 4001- 0401 | 1 | 1 | 2 |
| 3101- 4101- 0301 | 1 | 1 | 2 |
| 3101- 4901- 0101 | 1 | 1 | 2 |
| 3201- 1402- 07 | 1 | 1 | 2 |
| 3201- 2705- 1201 | 1 | 1 | 2 |
| 3201- 3501- 0101 | 1 | 1 | 2 |
| 3201- 3501- 0402 | 1 | 1 | 2 |
| 3201- 3501- 1305 | 1 | 1 | 2 |
| 3201- 4002- 1101 | 1 | 1 | 2 |
| 3201- 4002- 1302 | 1 | 1 | 2 |
| 3201- 4002- 1601 | 1 | 1 | 2 |
| 3201- 4402- 1301 | 1 | 1 | 2 |
| 3201- 4403- 1201 | 1 | 1 | 2 |
| 3303- 1516- 0102 | 1 | 1 | 2 |
| 6601- 4101- 1302 | 1 | 1 | 2 |
| 6801- 3505- 0404 | 1 | 1 | 2 |
| 6801- 3901- 0101 | 1 | 1 | 2 |
| 6801- 4402- 1101 | 1 | 1 | 2 |
| 6801- 5802- 1201 | 1 | 1 | 2 |
| 6802- 4403- 07 | 1 | 1 | 2 |
| 6802- 5301- 0801 | 1 | 1 | 2 |
| 0101- 4402- 1301 | 1 | 0 | 1 |
| 0101- 5701- 1301 | 1 | 0 | 1 |
| 0201- 1401- 07 | 1 | 0 | 1 |
| 0201- 1501- 0301 | 1 | 0 | 1 |
| 0201- 1501- 1501 | 1 | 0 | 1 |
| 0201- 1503- 0401 | 1 | 0 | 1 |
| 0201- 3502- 1104 | 1 | 0 | 1 |
| 0201- 3801- 1301 | 1 | 0 | 1 |
| 0201- 3906- 0801 | 1 | 0 | 1 |
| 0201- 4001- 07 | 1 | 0 | 1 |
| 0201- 4001- 1501 | 1 | 0 | 1 |
| 0201- 4002- 1101 | 1 | 0 | 1 |
| 0201- 4402- 1103 | 1 | 0 | 1 |
| 0205- 5001- 07 | 1 | 0 | 1 |
| 0301- 0702- 07 | 1 | 0 | 1 |
| 0301- 4402- 1201 | 1 | 0 | 1 |
| 1101- 2705- 0404 | 1 | 0 | 1 |
| 1101- 3501- 0301 | 1 | 0 | 1 |
| 1101- 3501- 1401 | 1 | 0 | 1 |
| 1101- 3701- 1501 | 1 | 0 | 1 |
| 2402- 0702- 1101 | 1 | 0 | 1 |
| 2402- 1401- 07 | 1 | 0 | 1 |
| 2402- 1501- 1301 | 1 | 0 | 1 |
| 2402- 3501- 1201 | 1 | 0 | 1 |
| 2402- 3801- 1104 | 1 | 0 | 1 |
| 2402- 4001- 0404 | 1 | 0 | 1 |
| 2402- 4402- 0401 | 1 | 0 | 1 |
| 2402- 4701- 0401 | 1 | 0 | 1 |
| 2601- 3801- 0401 | 1 | 0 | 1 |
| 2601- 4402- 1501 | 1 | 0 | 1 |
| 2603- 1501- 1501 | 1 | 0 | 1 |
| 2902- 4403- 0402 | 1 | 0 | 1 |
| 2902- 5801- 0804 | 1 | 0 | 1 |
| 3201- 0702- 07 | 1 | 0 | 1 |
| 3201- 1517- 1302 | 1 | 0 | 1 |
| 3201- 4402- 1201 | 1 | 0 | 1 |
| 3201- 4403- 0401 | 1 | 0 | 1 |
| 3201- 4901- 0101 | 1 | 0 | 1 |
| 3201- 5201- 1502 | 1 | 0 | 1 |
| 3201- 5501- 1501 | 1 | 0 | 1 |
| 6801- 5101- 1301 | 1 | 0 | 1 |
| 6802- 1402- 1303 | 1 | 0 | 1 |
| 0101- 0702- 1301 | 0 | 1 | 1 |
| 0101- 0801- 0404 | 0 | 1 | 1 |
| 0101- 0801- 1301 | 0 | 1 | 1 |
| 0101- 3701- 07 | 0 | 1 | 1 |
| 0101- 3701- 1301 | 0 | 1 | 1 |
| 0101- 3906- 0801 | 0 | 1 | 1 |
| 0101- 4402- 0401 | 0 | 1 | 1 |
| 0101- 4402- 0801 | 0 | 1 | 1 |
| 0101- 4901- 0101 | 0 | 1 | 1 |
| 0101- 5701- 10 | 0 | 1 | 1 |
| 0101- 5701- 1104 | 0 | 1 | 1 |
| 0101- 5701- 1501 | 0 | 1 | 1 |
| 0201- 0702- 07 | 0 | 1 | 1 |
| 0201- 0801- 0101 | 0 | 1 | 1 |
| 0201- 0801- 1301 | 0 | 1 | 1 |
| 0201- 1302- 0402 | 0 | 1 | 1 |
| 0201- 1401- 1501 | 0 | 1 | 1 |
| 0201- 1501- 0102 | 0 | 1 | 1 |
| 0201- 1501- 07 | 0 | 1 | 1 |
| 0201- 1503- 07 | 0 | 1 | 1 |
| 0201- 2705- 0401 | 0 | 1 | 1 |
| 0201- 3502- 0401 | 0 | 1 | 1 |
| 0201- 4001- 0101 | 0 | 1 | 1 |
| 0201- 4001- 1103 | 0 | 1 | 1 |
| 0201- 4403- 0301 | 0 | 1 | 1 |
| 0201- 4403- 0401 | 0 | 1 | 1 |
| 0201- 5101- 1301 | 0 | 1 | 1 |
| 0201- 5701- 1305 | 0 | 1 | 1 |
| 0205- 3901- 07 | 0 | 1 | 1 |
| 0301- 0702- 0401 | 0 | 1 | 1 |
| 0301- 1401- 07 | 0 | 1 | 1 |
| 0301- 1501- 0301 | 0 | 1 | 1 |
| 0301- 3501- 0401 | 0 | 1 | 1 |
| 0301- 4002- 1101 | 0 | 1 | 1 |
| 0301- 4402- 1501 | 0 | 1 | 1 |
| 0301- 4403- 07 | 0 | 1 | 1 |
| 0301- 4701- 1303 | 0 | 1 | 1 |
| 0302- 5001- 1101 | 0 | 1 | 1 |
| 1101- 0702- 1101 | 0 | 1 | 1 |
| 1101- 0702- 1301 | 0 | 1 | 1 |
| 1101- 2705- 1302 | 0 | 1 | 1 |
| 1101- 3701- 07 | 0 | 1 | 1 |
| 1101- 3801- 0402 | 0 | 1 | 1 |
| 1101- 4402- 1201 | 0 | 1 | 1 |
| 1101- 4402- 1401 | 0 | 1 | 1 |
| 1101- 5201- 1502 | 0 | 1 | 1 |
| 2301- 0702- 07 | 0 | 1 | 1 |
| 2301- 4403- 0401 | 0 | 1 | 1 |
| 2402- 1501- 1501 | 0 | 1 | 1 |
| 2402- 2502- 0401 | 0 | 1 | 1 |
| 2402- 3501- 1501 | 0 | 1 | 1 |
| 2402- 3801- 1305 | 0 | 1 | 1 |
| 2402- 4001- 0301 | 0 | 1 | 1 |
| 2402- 4402- 0101 | 0 | 1 | 1 |
| 2402- 4701- 1501 | 0 | 1 | 1 |
| 2402- 5601- 1501 | 0 | 1 | 1 |
| 2501- 1801- 1201 | 0 | 1 | 1 |
| 2601- 3801- 1104 | 0 | 1 | 1 |
| 2601- 4402- 07 | 0 | 1 | 1 |
| 2601- 4403- 0402 | 0 | 1 | 1 |
| 2603- 1501- 1201 | 0 | 1 | 1 |
| 2902- 1501- 0401 | 0 | 1 | 1 |
| 2902- 3801- 07 | 0 | 1 | 1 |
| 2902- 4403- 0804 | 0 | 1 | 1 |
| 2902- 5801- 07 | 0 | 1 | 1 |
| 3001- 3801- 1301 | 0 | 1 | 1 |
| 3002- 0702- 0404 | 0 | 1 | 1 |
| 3002- 1801- 1501 | 0 | 1 | 1 |
| 3101- 4001- 0301 | 0 | 1 | 1 |
| 3101- 4001- 07 | 0 | 1 | 1 |
| 3201- 0702- 0404 | 0 | 1 | 1 |
| 3201- 0801- 0301 | 0 | 1 | 1 |
| 3201- 1402- 1302 | 0 | 1 | 1 |
| 3201- 1517- 0101 | 0 | 1 | 1 |
| 3201- 3501- 1401 | 0 | 1 | 1 |
| 3201- 4001- 1302 | 0 | 1 | 1 |
| 3201- 5501- 0301 | 0 | 1 | 1 |
| 3201- 5501- 1201 | 0 | 1 | 1 |
| 3301- 1402- 0401 | 0 | 1 | 1 |
| 6801- 5101- 10 | 0 | 1 | 1 |
| 6802- 1402- 07 | 0 | 1 | 1 |
